# First-line Usage of Daratumumab, Lenalidomide, Dexamethasone (DRd) Combination in a Case of Castleman Disease Variant of Polyneuropathy, Organomegaly, Endocrinopathy, Monoclonal Gammopathy, and Skin Changes Syndrome (CD-POEMS)

**DOI:** 10.1097/HS9.0000000000000728

**Published:** 2022-06-17

**Authors:** Omur Gokmen Sevindik, Yasa Gul Mutlu, Berrin Balik Aydin, Istemi Serin

**Affiliations:** 1 Department of Hematology, Faculty of Medicine, Istanbul Medipol University, Turkey; 2 Department of Hematology, University of Health Sciences, Istanbul Training and Research Hospital, Turkey

Polyneuropathy, organomegaly, endocrinopathy, monoclonal gammopathy, and skin changes syndrome (POEMS) is a rare paraneoplastic syndrome of an underlying plasma cell disorder (PCD).^1^ Castleman disease (CD), itself, can also cause symptoms or signs of polyneuropathy, volume overload, cytokine release, but only those with peripheral neuropathy and a plasma cell clone can be classified as classical POEMS.^1^

Both classical POEMS syndrome with CD and CD variant of POEMS syndrome (CD-POEMS),^2^ literature lacks a standardized treatment approach. Here, we present a case who is diagnosed with CD-POEMS and successfully treated with a combination of daratumumab, lenalidomide, and dexamethasone (DRd).

A 54-year-old man had admitted to our department of neurology with weakness of both arms and legs. Three years ago, patient developed tingling on legs and ataxia. He needed assistance for walking. The neurological examination showed bilateral upper and lower limb muscle weakness especially dominant on distal parts of the extremities on admission. There was an evident atrophy on distal part of the extremities. Stocking-glove pattern for loss of vibration and pinprick sensation was found. Romberg sign was present. The rest of the neurological examination was normal. Electrodiagnostic study showed that sensory nerve action potential, compound muscle action potential is diminished on bilateral upper and lower extremities. To exclude a possible accompanying PCD, serum protein electrophoresis, serum immunofixation electrophoresis, serum free kappa, and lambda light chains were ordered and revealed an IgA/lambda monoclonal band. The amount of M protein was 0.23 g/dL and IgA was 559 mg/dL. A bone marrow biopsy was applied and showed a presence of 5% plasma cells with no clonality. The free light chain ratio was 1.5 at the time of diagnosis. Because of a previous computed tomography scan showing multiple mediastinal masses, a positron emission tomography (PET) scan was ordered to document any fluorodeoxyglucose (FDG) avid masses. Serum vascular endothelial growth factor (VEGF) level was 531 pg/mL (<96 pg/mL) and serum interleukin-6 (IL-6) level was measured as 24.75 pg/mL (<7 pg/mL) PET scan revealed axillary, paraaortic, and aortocaval masses with a biggest dimension of 5 × 4 cm and a maximum standardized uptake value of 3.5. Excisional biopsy of the most FDG avid mass was reported as hyaline vascular type CD. He also suffered from coronary artery disease and a chronic subdural hematoma, which are now regarded as the vascular complications of PCD. The patient was diagnosed as CD-POEMS and a combination of DRd was initiated with a protocol of daratumumab 16 mg/kg, weekly for cycles 1–2, biweekly for cycles 4–6, monthly for cycle 7 onwards; lenalidomide 25 mg, days 1–21 every 4 weeks; and dexamethasone 40 mg, weekly. The combination was well tolerated. After the first cycle of the treatment, a VEGF response was achieved and serum VEGF level was declined to 27.2 pg/mL. Also, the serum IL-6 level was decreased and measured as 6.31 pg/mL (Figure 1). The patient was able to stand up and walk with the help of 2 walking sticks. The PET scan revealed a complete response regarding the Castleman component of the disease. At the third cycle of DRd combination, he also underwent a successful revascularization of coronary arteries for the treatment of coronary artery disease. He continues the treatment with an ongoing and even deepening hematological and neurological response and able to walk without any support.

**Figure 1.**
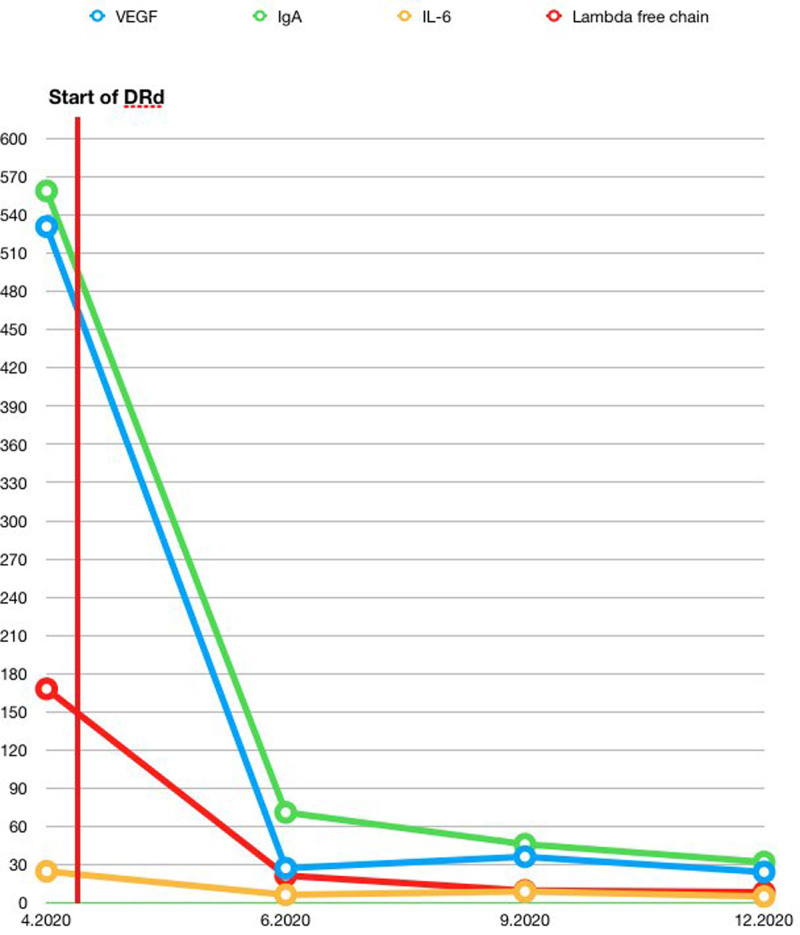
**Serum levels of VEGF, IgA, lambda free chain, and IL-6 after the start of DRd VEGF (pg/mL), IgA (mg/dL), IL-6 (pg/mL), and lambda free chain (mg/L).** DRd = daratumumab, lenalidomide, and dexamethasone; IL-6 = interleukin-6; VEGF = vascular endothelial growth factor.

There are 3 separate articles with similar characteristics in literature. Khan et al^3^ mentioned the use of DRd in a 60-year-old female patient diagnosed with POEMS in the second relapse that developed 18 months after autologous stem cell transplantation. Treatment of the patient who did not respond to lenalidomide (15 mg, days 1–21 and 28), ixazomib (4 mg, days 1, 8, and 15), and dexamethasone (20 mg weekly) was switched to DRd therapy and hematological-clinical complete response was achieved. In another case report, Gavriatopoulou et al^4^ preferred upfront DRd in 2 separate POEMS cases; also, clinical and hematological complete response was obtained. Tiew et al^5^ also mentioned the success of single-agent daratumumab usage in a relapsed/refractory POEMS case in their newly published article. The most important feature of our case was that it was the third one in “upfront usage” and the “first” among CD-POEMS cases.

Established risk factors for worse outcome in POEMS include low serum albumin, age, pleural effusion, pulmonary hypertension, and low glomerular filtration.^1^ In risk-adapted therapy, irradiation is the first-line treatment for a single plasmacytoma; systemic therapy is preferred in patients with diffuse sclerotic lesions or disseminated bone marrow involvement and progression 3–6 weeks after radiotherapy. Low-dose conventional therapy or high-dose stem cell transplantation forms the basis of treatment. Other treatment options are lenalidomide, thalidomide, and bortezomib with a manageable toxicity profile. For POEMS, cases not included in POLLUX^6^ and MAIA,^7^ the major studies that demonstrated the superiority of DRd compared with lenalidomide and dexamethasone in multiple myeloma, DRd is considered as a very promising therapy choice.

## ACKNOWLEDGMENTS

We respectfully remember all the colleagues we lost in the fight against COVID-19.

## AUTHOR CONTRIBUTIONS

All authors contributed to the editing of the article. IS prepared the accompanying figure.

## DISCLOSURES

Deva Holding A.S. was the sponsor for article processing charge. The authors have no conflicts of interest to disclose.

## ETHICS APPROVAL AND CONSENT TO PARTICIPATE

Written informed consent was obtained from the patient for publication of this report and any accompanying images.

## References

[1] Dispenzieri A. POEMS syndrome: 2021 update on diagnosis, risk-stratification, and management. Am J Hematol. 2021;96:872–888. 34000085 10.1002/ajh.26240

[2] Dispenzieri A. Castleman disease. Cancer Treat Res. 2008;142:293–330. 18283792 10.1007/978-0-387-73744-7_13

[3] Khan M Stone K van Rhee F. Daratumumab for POEMS syndrome. Mayo Clin Proc. 2018;93:542–544. 10.1016/j.mayocp.2018.02.001 29622102

[4] Gavriatopoulou M Ntanasis-Stathopoulos I Fotiou D. Upfront daratumumab with lenalidomide and dexamethasone for POEMS syndrome. HemaSphere. 2020;4:e381. 32647800 10.1097/HS9.0000000000000381 PMC7306302

[5] Tiew HW Sampath VS Gallardo CA. Single-agent daratumumab for refractory POEMS syndrome. Am J Hematol. 2022 March 7. [Online ahead of print]. 10.1002/ajh.26517 35253931

[6] Dimopoulos MA San-Miguel J Belch A. Daratumumab plus lenalidomide and dexamethasone versus lenalidomide and dexamethasone in relapsed or refractory multiple myeloma: updated analysis of POLLUX. Haematologica. 2018;103:2088–2096. 30237262 10.3324/haematol.2018.194282 PMC6269302

[7] Facon T Kumar SK Plesner T. Daratumumab, lenalidomide, and dexamethasone versus lenalidomide and dexamethasone alone in newly diagnosed multiple myeloma (MAIA): overall survival results from a randomised, open-label, phase 3 trial. Lancet Oncol. 2021;22:1582–1596. 34655533 10.1016/S1470-2045(21)00466-6

